# Screening for chronic conditions with reproductive factors using a machine learning based approach

**DOI:** 10.1038/s41598-020-59825-3

**Published:** 2020-02-18

**Authors:** Siyu Tian, Weinan Dong, Ka Lung Chan, Xinyi Leng, Laura Elizabeth Bedford, Jia Liu

**Affiliations:** 1 Department of Obstetrics and Gynecology, Li Ka Shing Faculty of Medicine, University of Hong Kong, Sassoon Road, Hong Kong, China; 2 Department of Family Medicine and Primary Care, Li Ka Shing Faculty of Medicine, University of Hong Kong, Hong Kong, China; 3 Shenzhen Institutes of Advanced Technology, Chinese Academy of Sciences, Shenzhen, China; 4 Department of Medicine and Therapeutics, Prince of Wales Hospital, The Chinese University of Hong Kong, Sha Tin, Hong Kong

**Keywords:** Population screening, Epidemiology

## Abstract

A large proportion of cases with chronic conditions including diabetes or pre-diabetes, hypertension and dyslipidemia remain undiagnosed. To include reproductive factors (RF) might be able to improve current screening guidelines by providing extra effectiveness. The objective is to study the relationships between RFs and chronic conditions’ biomarkers. A cross-sectional study was conducted. Demographics, RFs and metabolic biomarkers were collected. The relationship of the metabolic biomarkers were shown by correlation analysis. Principal component analysis (PCA) and autoencoder were compared by cross-validation. The better one was adopted to extract a single marker, the general chronic condition (GCC), to represent the body’s chronic conditions. Multivariate linear regression was performed to explore the relationship between GCC and RFs. In total, 1,656 postmenopausal females were included. A multi-layer autoencoder outperformed PCA in the dimensionality reduction performance. The extracted variable by autoencoder, GCC, was verified to be representative of three chronic conditions (AUC for patoglycemia, hypertension and dyslipidemia were 0.844, 0.824 and 0.805 respectively). Linear regression showed that earlier age at menarche (OR = 0.9976) and shorter reproductive life span (OR = 0.9895) were associated with higher GCC. Autoencoder performed well in the dimensionality reduction of clinical metabolic biomarkers. Due to high accessibility and effectiveness, RFs have potential to be included in screening tools for general chronic conditions and could enhance current screening guidelines.

## Introduction

Type 2 diabetes mellitus (T2DM), hypertension and hyperlipidemia are chronic conditions that can result in severe complications^1,2^, including cardiovascular disease (CVD), the leading cause of death worldwide^3,4^. Unfortunately, a large number of patients with these conditions remain undiagnosed. Most updated literatures showed that in 2019 there are 50.1% (231.9 million) of diabetes patients still undiagnosed worldwide^5^. A large proportion of cases with hypertension^6^ and hyperlipidemia^7^ are also unware of their condition, particularly in low and middle income countries^8^.

Screening of those at risk of chronic conditions is of significance for both individuals and wider society, yet there are gaps in current practices. Early identification is generally based on commonly collected risk factors, such as age, gender, smoking status, body mass index (BMI) and family history. A number of societies and task forces have recommended various screening guidelines that consist of these risk factors^9–11^; however, there are growing concerns that such guidelines might be inadequate and inaccurate^12–15^. For example, the American Diabetes Association (ADA) and the US Preventive Services Task Force (USPSTF) guidelines have shown only a fair performance when externally validated^12,13^. Furthermore, a trial exploring the effectiveness of a population-based screening programme in the United Kingdom found that screening was not associated with a reduction in all-cause mortality over a median period of 9.6 years^15^. A number of commonly used screening functions have also been shown to be ineffective in population screening^16^.

To include novel or extra factors might help to identify high risk groups more accurately and has the potential to improve current screening guidelines for chronic conditions, in terms of both effectiveness and efficiency. Indeed, a growing body of studies has identified a strong relationship between women’s reproductive factors (RF) and chronic conditions. For example, early menarche has been found to be associated with an increased risk of T2DM^17,18^, obesity and insulin resistance^19^. Moreover, a retrospective study conducted in Europe showed that, after adjustment for confounding, early menopause and shorter reproductive life span was associated with T2DM^20^. A Japanese study also found a similar relationship regarding hypercholesterolemia^21^. Furthermore, the China Kadoorie Biobank study reported that Chinese women with late menopause (≥53 years) were 1.21 (95% CI: 1.03–1.42) times more likely to have T2DM than women with menopause at 46–52 years old (p < 0.0001)^22^. Another Chinese study also found that a higher number of live births was associated with hypertension and DM, and mediated by lifestyle and dyslipidemia^23^. Additional studies conducted in different regions and healthcare settings have shown similar results. It is also important to note that RFs are highly accessible in all medical settings with low cost, hence we hypothesized that RFs are associated with chronic conditions and, as novel factors, might be able to improve current screening guidelines to assess women’s risk of chronic conditions^21^.

The objective of the current study is to explore the relationship between RFs and chronic conditions in order to assess the application of RFs as preliminary screening tools for general chronic conditions in women, so as to allow for the early diagnosis and intervention. This is challenging as clinical biomarkers of chronic conditions consist of multiple parameters as dependent variable and it is difficult to clarify its relationship with RFs using standard statistical methods. Therefore, in order to investigate their association, we applied a machine learning based dimensionality reduction technique, autoencoder, to generalize one single marker to represent chronic conditions.

## Methods

### Participants

A cross-sectional study was conducted in the Gansu Province of China. Random stratified sampling was adopted to include participants who were under care in primary health service organizations. The sample size was assessed by the following formula^24^: ${\rm{N}}=\frac{{{\rm{Z}}}_{\frac{{\rm{\alpha }}}{2}}^{2}{\rm{P}}1-{\rm{P}}}{{{\rm{\varepsilon }}}^{2}}$. It was estimated that 384 participants ought to be enrolled from each of the 28 sample centres (accuracy = 95%, confidence = 95%). To allow for missing data, and the constitution of demographic factors, 12,000 participants were recruited, of which 11,115 completed the study.

Participants were eligible for inclusion if they were: (1) female; (2) postmenopausal; (3) no self-reported DM, hypertension or dyslipidemia.

Participants were excluded if they were: (1) diagnosed with secondary diabetes or secondary hypertension; (2) pregnant and lactating; (3) taking medicine that affects the metabolism of glucose and lipids within 3 months; and (4) diagnosed with type 1 diabetes; (5) Non-natural menopause.

### Study design

From June to August 2016, seven investigators with a registered nursing practicing certificate administered a questionnaire, physical tests and biochemical tests to participants.

The questionnaire was designed based on related studies^25,26^ and modified according to pre-survey results in order to minimize bias. The investigators interviewed each participant face-to-face and completed the questionnaire accordingly. Five RFs were collected: age at menarche, age at menopause, reproductive life span, live births and abortion history.

The investigators performed five physical tests using standard instruments to measure height, weight, waist and hip circumference, heart rate, systolic blood pressure (SBP) and diastolic blood pressure (DBP). All tests were repeated three times and the average reading calculated. Body mass index (BMI) was calculated with weight (kg)/height^2^ (m^2^), and waist-to-hip ratio (WHR) was calculated using waist (cm)/hip (cm).

Three biochemical tests were performed, which included: 1) a fast blood-glucose test; 2) a blood lipids test; and 3) oral glucose tolerance test (OGTT). All laboratory assays were performed in accredited medical laboratories by the Chinese National Health Authority. Protocols were strictly adhered to. Total cholesterol (TC), total triglyceride (TG), high-density lipoprotein (HDL-C), low-density lipoprotein (LDL-C), fasting plasma glucose (FPG), OGTT 2 h plasma glucose (OGTT 2 h PG) were collected.

### Ethical considerations

The ethics committee of the School of Public Health in Lanzhou University approved this study. All relevant ethical guidelines and regulations were strictly adhered to throughout. Informed consent was obtained from each participant whose data was included in the analyses.

### Data analysis

First, a descriptive analysis was applied to summarize the biomarkers and RFs. Following that, since all the clinical biomarkers are continuous variables and are verified following normal (Gaussian) distribution (by Kolmogorov-Smirnov normality test), the relationship between included clinical biomarkers was explored using Pearson correlation analysis and hierarchical clustering analysis. Corresponding correlation plots were used to display the complex relationships between each of the two variables^27^. Through this method, we were able to demonstrate the redundancy of the clinical biomarkers. An autoencoder was then applied to generalize a single marker to represent 10 clinical biomarkers of the chronic conditions. Meanwhile, a more generic dimensionality reduction method, principle component analysis (PCA), was applied for comparison. Disease binary variables (positive or negative, represented by 1 or 0) of pathoglycemia, hypertension and dyslipidemia were determined from the continuous values of the original 10 clinical biomarkers according to the clinical ascertainment of these diseases. For both methods, using disease binary variables as labels and extracted single variable as the risk score to set different threshold, area under curve (AUC) with 95% confidence interval was calculated based on 10-fold cross-validation, in order to verify the representation power of the extracted variable. T-test was used to compare the representation power (AUC) of both methods.

An autoencoder is a data-driven neural network with an encoder, a bottleneck layer and a decoder combined. The encoder (also a multilayer network) can project the high-dimensional data onto a low-dimensional feature space at the output of the bottleneck layer, which can also be considered as a feature extraction of the input. The multilayer decoder network can then reconstruct the data from the coder layer reversely. Therefore, the network can be trained unsupervised with the same input and output by minimizing the mean square error (MSE) between them at the output of the network using a backpropagation algorithm^28^. The bottleneck layer is considered as a valid dimensionality reduction or feature extraction and then it can be used as a generalized marker to represent the input (the 10 biomarkers).

**Figure 1:**
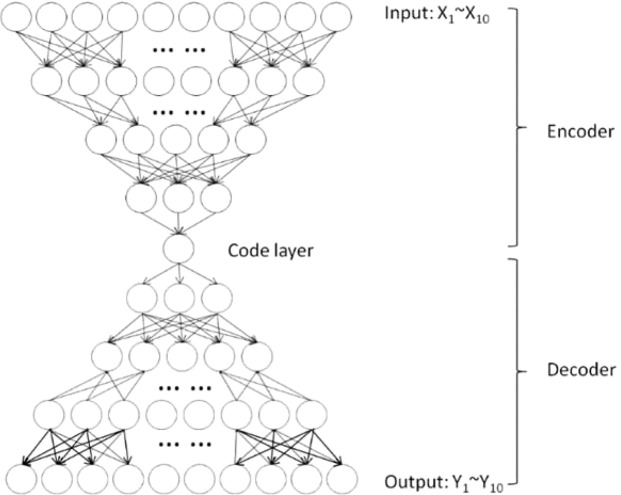
Structure of the multilayer autoencoder.

The structure of this autoencoder is presented in Fig. 1. Ten clinical biomarkers were used as the inputs and outputs to train the final autoencoder. All activation functions were set to be sigmoid functions for nonlinear transformation. Initially, considering the sample size and degree of freedom (decided by the number of parameters to be estimated), we set up a range for the number of layers and neurons. The specific numbers of layers and neurons were finally determined by greedy search, as well as other hyper-parameters, such as the learning rate and batch size.

After the better dimensionality reduction method was identified, it was used to extract the single biomarker of all cases. This single general marker is named as the general chronic condition (GCC) in this paper. Receiver operating characteristic curves (ROC) were plotted to show the capability of GCC to represent these three chronic conditions.

The relationship of GCC and RFs was then analyzed using multivariate linear regression. This confirmed whether the reproductive factors were associated with the GCC, and in other words, whether they are effective preliminary screening tools for chronic conditions. Missing data were handled using multiple imputation by chained equation (MICE) for 5 times and the results were pooled with Rubin’s rule^29^.

Statistical analysis was implemented on R 3.5.1. All significance tests were two-tailed and α = 0.05. PCA was implemented by *prcomp* function, and AUC was calculated by *pROC* package. The autoencoder was implemented on Python 3.5.4 using Tensorflow, which is an open-source software library for machine learning. Raw dataset and code can be found on Github. (https://github.com/dongdongdongdwn/Reproductive-factors-as-screening-tools-for-chronic-conditions-in-primary-care-using-a-machine-learn).

## Results

### Participant characteristics

**Figure 2:**
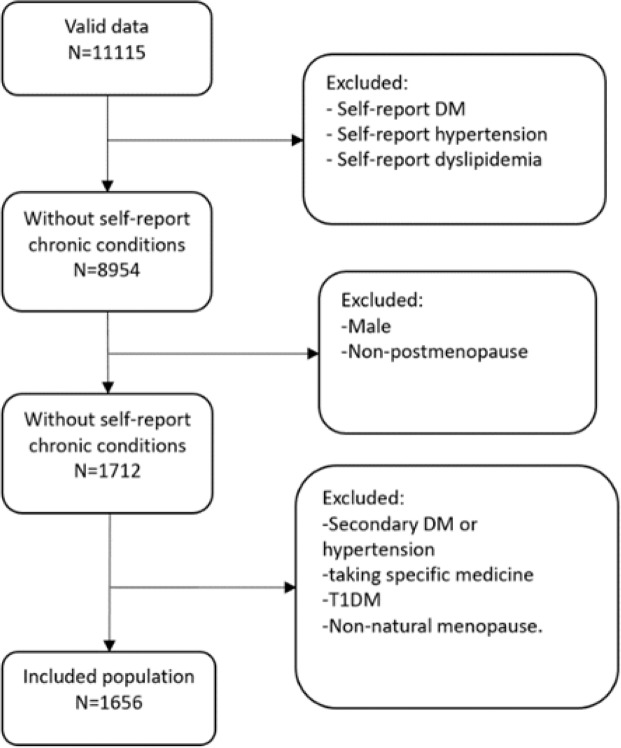
Flow chart of included population.

As shown in Fig. 2, 11,115 cases with valid data were included initially. According to our inclusion and exclusion criteria, 1,656 postmenopause women without self-report chronic conditions were retained for further analysis.

The clinical biomarkers collected from physical and biochemical tests are listed in Table 1 with respect to three age groups (years: 41–50, 51–65, >65). The mean values of the BMI were statistically different (*p* < 0.01) across the groups. A similar finding was also apparent for WHR, TC, fasting plasma glucose, OGTT 2 h plasma glucose, SBP and DBP. Table 2 describes the distribution of the 5 reproductive factors.

**Table 1:** Clinical biomarkers by categories of age (N = 1656, mean ± std).

Biomarkers	Total	Age			
		41–50 (n = 577)	51–65 (n = 554)	>65 (n = 525)	*P* value
BMI(kg/m^2^)	22.54 (2.91)	22.85 (2.56)	23.37 (2.79)	22.98 (3.32)	<0.01
WHR	0.86 (0.07)	0.86 (0.06)	0.86 (0.07)	0.87 (0.07)	<0.05
TC(mmol/L)	4.46 (0.94)	4.46 (0.94)	4.50 (0.87)	4.55 (1.01)	<0.05
TG(mmol/L)	1.73 (1.16)	1.64 (1.13)	1.80 (1.18)	1.73 (1.13)	0.23
HDL-C(mmol/L)	1.30 (0.31)	1.32 (0.27)	1.29 (0.30)	1.32 (0.35)	0.24
LDL-C(mmol/L)	2.75 (0.72)	2.72 (0.70)	2.79 (0.65)	2.72 (0.77)	0.19
FPG(mmol/L)	4.88 (0.90)	4.80 (0.74)	4.99 (0.91)	5.13 (1.21)	<0.01
OGTT 2 h PG(mmol/L)	6.50 (2.10)	6.70 (2.18)	6.99 (2.28)	7.11 (2.48)	<0.01
SBP(mmHg)	120.39 (13.75)	118.00 (11.55)	124.36 (12.89)	129.39 (16.22)	<0.01
DBP(mmHg)	76.50 (11.76)	76.03 (7.24)	77.93 (15.20)	80.67 (16.13)	<0.01

(Note: Levene’s Test showed the variances were statistically equal between groups (p < 0.05) for each variable, one-way ANOVA was adopted to examine the difference between groups; Abbreviation: BMI = body mass index; WHR = waist-hip-ratio; TC = total cholesterol; TG = triglyceride; HDL-C = high density lipoprotein cholesterol; LDL-C = low density lipoprotein cholesterol; FPG = fasting plasma glucose; OGTT 2 h PG = OGTT 2 hour plasma glucose; SBP = systolic blood pressure; DBP = diastolic blood pressure).

**Table 2:** The distribution of the reproductive factors (N = 1656).

Reproductive Factors		n (%)
Age at menarche	≤12	77 (4.65%)
	13	387 (23.37%)
	14	662 (39.98%)
	15	219 (13.22%)
	16	129 (7.79%)
	≥17	182 (10.99%)
Age at menopause	≤45	137 (8.27%)
	46–48	477 (28.8%)
	49–50	691 (41.73%)
	≥51	353 (21.32%)
Live births	0	200 (12.08%)
	1	752 (45.41%)
	2	468 (28.26%)
	≥3	236 (14.25%)
Abortion history	0	1532 (92.51%)
	1	86 (5.19%)
	≥2	39 (2.36%)
Reproductive life span	≥40	108 (6.52%)
	37–39	272 (16.43%)
	34–36	641 (38.71%)
	30–33	452 (27.29%)
	≤29	182 (10.99%)

### Correlation within clinical biomarkers

**Figure 3:**
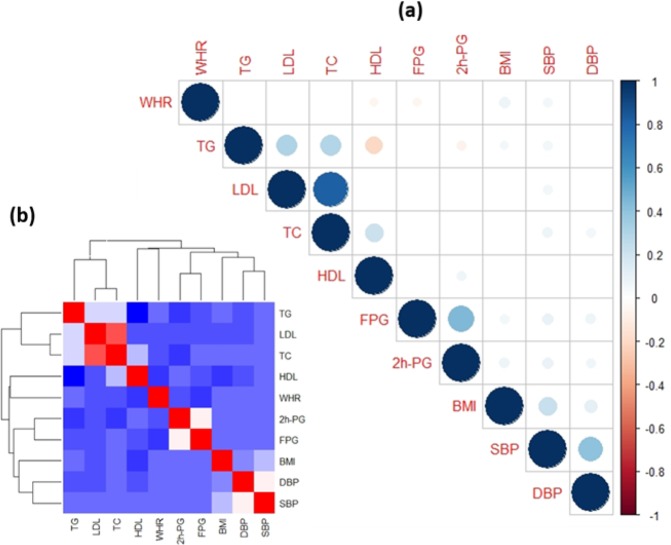
Correlation matrix and Hierarchical Clustering of the clinical biomarkers (**a**) Correlation matrix of biomarkers; (**b**) Hierarchical clustering.

According to the correlation matrix and Hierarchical Clustering of the 10 clinical biomarkers (Fig. 3), none of the biomarkers were uncorrelated, which implies that complicated relationships and strong redundancy exist across the biomarkers, and dimensionality reduction could potentially extract a better representation.

### Dimensionality reduction

Due to the internal correlation within the biomarkers, a dimensionality reduction method is reasonable to be used to extract representative features. Multilayer autoencoder and PCA were performed based on 10-fold cross validation and results were compared with a t-test. As shown in Table 3, AUCs of autoencoder were significantly higher (p value < 0.01) than the PCA for all chronic conditions (pathoglycemia, hypertension, dyslipidemia), indicating that autoencoder could produce a more representative variable to express the body’s metabolism.

**Table 3:** Comparison of the discrimination power (AUC) of extracted factors by autoencoder and PCA.

	Autoencoder	PCA	p value
Pathoglycemia	0.827 (0.814–0.838)	0.569 (0.560–0.579)	<0.01
Hypertension	0.809 (0.794–0.821)	0.662 (0.651–0.674)	<0.01
Dyslipidemia	0.801 (0.788–0.813)	0.674 (0.669–0.679)	<0.01

(Note: AUCs with 95% CI are reported, t-test is used to examine the statistical difference).

**Figure 4:**
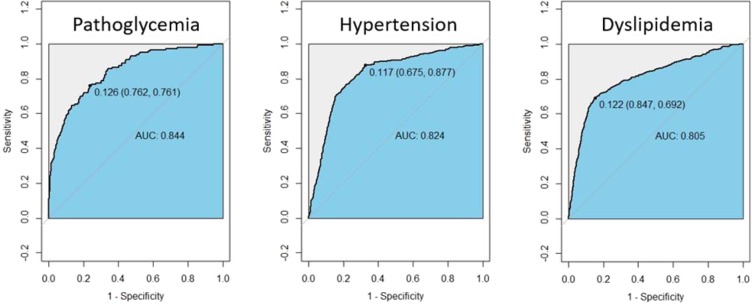
Discrimination power of GCC for different chronic conditions Area under ROC curve is adopted. The optimal thresholds to distinguish positive and negative cases were presented.

Subsequently, autoencoder was applied to all cases, translated 10 biomarkers into a single general marker (GCC). As shown in Fig. 4, when distinguishing pathoglycemis, hypertension and dyslipidemia respectively, GCC showed good discrimination power with an AUC of more than 0.8 (AUC = 0.844, 0.824, 0.805).

### Association between the reproductive factors and the GCC

Finally, the relationship between RFs and GCC was explored by multivariate linear regression. As illustrated in Table 4, after adjustment of age, GCC, as the dependent variable, was associated with age at menarche (p < 0.05) and reproductive life span (p < 0.01). It was also found that GCC was higher with early age at menarche (OR = 0.9976, 95%CI: 0.9961–0.9998) and shorter reproductive life span (OR = 0.9895, 95%CI: 0.9926–0.9864). No significant results were found as for age at menopause, live births and abortion history.

**Table 4:** Relationship between RFs and GCC (N = 1656).

Independent variables	OR	*p*	OR 95% CI	
			Lower	Upper
Age at menarche	0.9976	0.0190*	0.9961	0.9998
Age at menopause	1.0026	0.0593	1.0015	1.0036
Reproductive life span	0.9895	0.0000*	0.9926	0.9864
Live births	1.0016	0.2088	0.9991	1.0041
Abortion history	0.9995	0.8846	0.9932	1.0059
Age	1.0000	0.4721	1.0000	1.0000
(Constant)	0.9883	0.7012	0.9303	1.0498

(Note: Multivariate linear regression with forward stepwise is used. *p < 0.05).

## Discussion

This study explored the relationship between chronic conditions and reproductive factors and demonstrated that age at menarche and reproductive life span have potential to be incorporated into screening tools for general chronic conditions. The chronic conditions were generalized from relevant clinical biomarkers using one of the most advanced non-linear dimensionality reduction techniques in machine learning. Autoencoder outperformed a state-of-the-art dimensionality reduction method, PCA, and extracted a more discriminative general marker. To our knowledge, there is currently no similar study reported.

Metabolic syndrome (MS) is a traditional way to represent the chronic conditions, but had been given considerable doubt by both the American Diabetes Association and the European Association regarding its value as a CVD risk marker as too much critically important information was missing to warrant its designation as a “syndrome”^30^. Meanwhile, it has been shown that MS is insensitive to identifying some chronic metabolic diseases^31^. However, it is well know that these chronic conditions (i.e. pathoglycemia, hypertension and dyslipidemia) are characterized by metabolic disorder and show clustering on account of similar risk factors and correlative physiological mechanisms^32,33^. Hence, we used a machine learning based approach, autoencoder, to generate a representative marker to represent these chronic conditions.

Machine learning and artificial intelligence have become emerging techniques in health care for big data analysis^34,35^. Autoencoder was first introduced by Hinton in 2006 and has been verified to outperform traditional approaches for dimensionality reduction^36^ and gained increasing use as an application in medical studies^37^. In this study, the autoencoder was trained with more than 1,000 samples with 10 biomarkers and found to successfully extract one single marker, the GCC, to generalize the biomarkers for the chronic conditions. GCC was also shown to have the power to discriminate the chronic diseases (AUCs > 0.8). In comparison, the marker that was extracted by PCA was not discriminative enough (AUCs < 0.7). This could be interpreted by the nonlinear expressiveness of multilayer autoencoder^36^ which derives from its multiple hidden layers and nonlinear activation functions^38^. The improved nonlinear reconstruction of autoencoder over PCA has also been verified and explained by other researchers^39,40^. In addition, it is well known that a multilayer autoencoder requires more computation than PCA, however, due to the limited sample size (N = 1,656), these two methods did not show apparent difference in the computation time.

Via the multivariate linear regression, earlier age at menarche and shorter reproductive life span have been found to be associated with chronic conditions. In terms of age at menarche, our findings are in accordance with numbers of studies in which females with early age at menarche are at higher risk of chronic diseases. A recent study on Chinese elderly women (age = 70.39 ± 6.21) reported that women with metabolic syndrome had younger menarche age, higher gravidity and parity^41^. Additionally, in a multicenter case control study, Lecinana and his colleagues determined that very early exposure onset (age < 13) may do harm to body metabolism function^42^. Generally, adulthood adiposity is considered as potential mediator^43^. Apart from that, the association between reproductive life span and the risk of chronic conditions is also supported by relevant studies and could be interpreted by the protective effect of estrogen^20^. In terms of the age at menopause, currently there is not a uniform conclusion as some studies have not found any relationship^44,45^ whereas some have^46,47^. Mechanistic studies have demonstrated beneficial effects of estrogen on insulin secretion and glucose homeostasis. Meanwhile, some researchers believe the effect on TC is a result of a decrease in serum estradiol^48^ and a decrease in the activity of LDL-C receptors^49^. There is also an assumption that insulin resistance is associated with pregnancy and parturition^50,51^. However, after multivariable adjustment, we did not observe any such association. Besides, some specific reproductive conditions, such as polycystic ovary syndrome (PCOS), are associated with insulin resistance^52^ and secondary hyperandrogenism^53^. Lagana and his colleagues also found that insulin sensitizers could improve the PCOS symptoms^54^, which hints that additional RFs could benefit the chronic conditions screening. In sum, despite the fact that the relationship between RFs and chronic conditions could not be interpreted by a single factor, RFs do have strong associations with chronic conditions.

Low cost disease detection models are of great importance to reduce the health economic burden, and especially to benefit developing countries^55^. High accessibility and low cost are outstanding advantages of RFs. Furthermore, there are studies that have shown that the validity and reproducibility of self-reported RFs are good^56^. Therefore, RFs have potential as screening tools for chronic conditions and could improve current screening guidelines.

A number of important limitations need to be considered. First, this is a cross-sectional study and hence it cannot infer causality. Second, this study only tested the possibility that RFs can be incorporated into a screening tool and did not give the actual sensitivity and specificity of RFs to screen for chronic conditions. In terms of further research, a structured screening tool should be developed and externally validated. Third, although not included in the current study, uric acid and HbA1c are also crucial biomarkers for chronic conditions and it is important that future research takes them into account. Last, interpretability is always a key concern when applying machine learning to medical data analysis. Many advanced methods have been proposed to unfold the black box of neuron networks. In future study, we hope to focus on this specific question and explore the GCC more comprehensively.

To conclude, autoencoder performed well in the dimensionality reduction of clinical biomarkers, demonstrating its potential in further medical data process. Women with earlier age at menarche and shorter reproductive life span are more likely to suffer from chronic conditions. Due to high accessibility and effectiveness, RFs show potential to be included in preliminary screening tools for general chronic conditions in clinical practice and could enhance current screening guidelines.

## Data Availability

The original data is not currently available online but can be requested in machine-readable format from the corresponding author on reasonable request.
